# Rat bite fever in a total knee arthroplasty: an unusual case of periprosthetic joint infection

**DOI:** 10.1186/s42836-022-00114-x

**Published:** 2022-04-02

**Authors:** Anne Theunis Fokkema, Linda Martine Kampschreur, Loredana Elena Pirii, Wierd Pieter Zijlstra

**Affiliations:** 1 Department of Orthopedic Surgery, Medical Center Leeuwarden, Henri Dunantweg 2, Leeuwarden, The Netherlands 8934 AD; 2 Department of Internal Medicine, Medical Center Leeuwarden, Leeuwarden, The Netherlands; 3 Certe Center For Infectious Diseases Friesland, Leeuwarden, The Netherlands

**Keywords:** Total knee arthroplasty, Prosthetic joint infection, Rat bite fever, DAIR, Streptobacillus *moniliformis*

## Abstract

**Background:**

Periprosthetic joint infection (PJI) is a serious complication of orthopedic arthroplasty surgery. Rat bite fever is a rare infection with *Streptobacillus moniliformis (S. moniliformis)*. Rat bite fever can lead to serious complications such as pyogenic infection of joints, bacteremia, endocarditis and even death. We hereby present the first case of a patient with a total knee arthroplasty, infected with *S. moniliformis,* successfully treated by surgical debridement, antibiotics and implant retention (DAIR).

**Case presentation:**

The patient was a 64-year-old female. *S. moniliformis* was isolated from blood cultures and an aspirate of the left knee by 16S rRNA gene polymerase chain reaction technique. It was assumed that the *S. moniliformis* had a systemic origin and secondarily infected the knee due to fever nine days before the onset of symptoms of the knee. The patient was successfully managed with DAIR and intravenous administration of ceftriaxone for six weeks and oral doxycycline for another six weeks.

**Conclusions:**

*S. moniliformis* is a rare pathogen and is difficult to culture. The 16S rRNA sequencing is helpful in the determination of a causative microorganism in the case of a culture-negative PJI. A DAIR procedure in combination with 12 weeks of antibiotics could successfully treat *S. moniliformis* prosthetic joint infection.

## Background

Periprosthetic joint infection (PJI) is a serious complication of orthopedic arthroplasty surgery. Common causative bacteria are *Staphylococcus aureus* and coagulase-negative *Staphylococci* [1]. Rat bite fever (RBF) is a rare zoonotic infection caused by *Streptobacillus moniliformis (S. moniliformis)* or *Spirillum minus* [2]. *S. moniliformis* is a gram-negative rod that was first isolated in 1914 by Schotmüller [3]. It colonizes the nasopharynx of rats and other rodents [4]. Patients can be infected by either a rat bite or scratch, or oral ingestion of substances soiled by rat feces and urine with *S. moniliformis* [2]. Approximately 30% of the patients cannot recall contact with a rat [5]. The disease typically presents itself with fever, pharyngitis, headache, arthralgias and skin rash [2]. Typically there is minimal inflammatory reaction over the site of infection [2]. Various complications of RBF are known, such as reactive arthritis or pyogenic septic arthritis, bacteremia, endocarditis, pneumonia, spondylodiscitis, osteomyelitis and metastatic abscess [2, 6, 7]. Recommended treatment may consist of surgical debridement and lavage of the septic joint. Based on limited experience, recommended antibiotic therapy should be five to seven days of intravenous penicillin, followed by seven days of oral medication [8]. *S. moniliformis* is known as an extremely fastidious bacterium that needs microaerophilic conditions to grow and this makes the diagnosis difficult [5, 9]. While arthralgia, pyogenic infection of the joint, antibiotic treatment of prosthesis infection and even arthroscopic treatment of an infected knee prosthesis have been reported before. We hereby present, to the best of our knowledge, the first case of a PJI caused by *S. moniliformis* in a patient who received a total knee arthroplasty (TKA) and was managed by surgical Debridement, Antibiotics and Implant Retention (DAIR) [10, 11].

## Case presentation

A 64-year-old female patient, with a medical history of axial spondylarthropathy, diabetes mellitus type II, hypertension, morbid obesity and laparoscopic Roux en Y bypass, received a left TKA in 2015 for end-stage osteoarthritis. The surgical procedure and follow-up were uneventful. In May 2021, the patient returned with pain in both shoulders, upper arms, the right wrist and, most prominently, in the left knee. She reported that she had felt ill for a day and had 39 degrees fever nine days before she visited the hospital. The physical examination showed a non-acute ill patient with a large swelling of the left knee, and a heavily impaired function with a flexion of 50 degrees, and extension deficit of 30 degrees. The blood examination showed a C-reactive protein (CRP) of 156 mg/L and a white blood cell count (WBC) of 13.1 × 10^9^/L per liter. A sterile puncture was performed and samples were taken and analyzed for gram stain, culture and uric crystals, and blood cultures were taken. A rheumatologist was consulted and the aspirate of the knee was analyzed for uric crystals, which were not found. The gram staining was negative but was positive for leukocytes. Initially, the clinical diagnosis was made of reactive arthritis due to systemic infection with an unknown primary location. The patient was hospitalized for close monitoring, while antibiotics were withheld. On the second day of admission, the blood cultures turned positive for a gram-negative rod. Intravenous cefuroxime was started at 1.5 g four times a day. Due to minor clinical improvement with a decrease in knee pain and a drop of CRP to 120 mg/L, no surgery was performed the second day. The third day after admission, the CRP, however, rose to 149 mg/L again, and the patient reported more pain in the knee. Therefore, the decision was made to perform a Debridement, Antibiotics and Implant Retention (DAIR) procedure. Preoperatively, two grams of cefazolin was administered to prevent secondary infections. An open arthrotomy was performed. Perioperatively, there was no collection of pus but cloudy synovium was observed. Four cultures were taken of synovial fluid, periprosthetic tissue, joint capsule and interface tissue around the prosthesis, each with a clean instrument. The prosthesis insert could not be removed due to unavailability of the insert because this type of prosthesis was discontinued by the manufacturer. If it had been available, the insert would have been exchanged for a new insert. Extensive debridement, with all “suspicious” tissues excised, was performed, by using a diathermic knife. The prosthesis and surrounding tissues were then brushed manually with a scrubbing sponge and povidone-iodine for at least two minutes. This was followed by extensive flushing of the knee by pulsating lavage with six liters of 0.9% saline solution. Subsequently, a new extremity drape was placed around the knee, and gloves were changed and the knee was closed in layers using new instruments. Immediately after surgery, the patient improved clinically. A trans-esophageal ultrasound was performed on the request of the infectious disease specialist and showed no evidence of infectious endocarditis. The blood cultures (BD Bactec) became positive after two incubation days and they were cultured aerobically and anaerobically on solid media. The bacteria were seen after five days on blood agar with 5% sheep blood and on chocolate agar media. A Vitek MS matrix-assisted laser desorption/ionization time-of-flight mass spectrometer (MALDITOF) (bioMérieux) was used based on mass spectrometry principle for the identification of the grown colonies, but the identification failed repeatedly. The identification with Vitek MS MALDITOF (bioMérieux) depends on the database of the analyzer which can impose limitations. For this reason, molecular diagnostics using the 16S rRNA gene polymerase chain reaction technique (PCR) were chosen to help the identification and eventually *S. moniliformis* was found. The knee aspirate was also cultured and after 7 days of incubation. *S. moniliformis* was also identified in the knee aspirate by 16S rRNA gene sequencing. Unfortunately, the susceptibility of *S. moniliformis* could not be determined despite all the efforts to use microaerophilic conditions and Schaedler anaerobe agar with sheep blood with haemin and vitamin K1 as advised in the literature [5]. Knowing the etiological agent of bacteremia, the antibiotics were switched to ceftriaxone based upon literature review [8]*.* Supplementary history taking was performed to trace the origin of the infection. The patient reported having no memory of a rat bite, nor living close to rats, nor living close to open water or having eaten spoiled food. She reported having two dogs. Possibly the dogs could have been infected by *S. moniliformis* by either eating rats, being bitten by rats or by swimming in water polluted by rat feces. Therefore, both dogs were swabbed and samples were taken of their saliva. *S. moniliformis* could not be cultured and recovered from the dogs. Sixteen days after the DAIR, the patient could be discharged home with intravenous antibiotics in good clinical condition. The CRP at time of discharge was 20 mg/L and the WBC was normal at 3.9 × 10^9^/L. A total antibiotic regimen of twelve weeks was planned according to clinical guidelines [12]. After multidisciplinary consultation with a medical microbiologist, an infectious diseases specialist and an orthopedic surgeon, the following antibiotic regimen was agreed upon: six weeks intravenous ceftriaxone two grams once daily because of bacteremia and another six weeks of oral doxycycline 200 mg once daily to complete the treatment of the PJI [12]. Due to penicillin allergy confirmed with skin testing, the patient could not be treated with oral amoxicillin, which normally would be the oral antibiotic of choice. At three, six and thirteen weeks after surgery, the patient was seen for follow-up. At the most recent follow-up at thirteen weeks when the patient was one week after the last administration of doxycycline, the pain had diminished. The function of the knee was improved to normal walking with a flexion of 120 degrees and full extension to zero degrees. Laboratory results showed CRP < 1 mg/L and WBC of 2.9 × 10^9^/L.

## Discussion

To the best of our knowledge, this is the first case of periprosthetic joint infection with *S. moniliformis* in a patient with a TKA, which was successfully treated by DAIR and a 12-week course of intravenous and oral antibiotics. The use of 16S rRNA sequencing was vital for establishing the diagnosis.

Rat bite fever is a rare zoonotic infection characterized by fever, rash and arthritis caused by *S. moniliformis* [2]. *Streptobacillus moniliformis* is a pleomorphic, filamentous, gram-negative, non-motile and non-acid fast bacillus. The bacteria grow slowly (2 to 3 days) and may take as long as 7 days to yield the positive result. The biofilm-forming capacity of *S. moniliformis* is still unknown and future research is necessary. *S. moniliformis* is an extremely fastidious bacterium that needs microaerophilic conditions to grow. This makes microbiological diagnosis difficult*.* Optimal growth requires Trypticase soy agar or broth enriched with 20% blood and microaerophilic conditions [5]. Once bacterial growth takes place, it is worthwhile to try to identify it by using a MALDITOF MS analyzer. This was not successful in this case. Nonetheless, we expect that, in the future, the databases of the analyzers will be extended with new bacterial profiles and identification of *S. moniliformis* can become possible. Until then, the 16S rRNA sequencing remains the fundamental method for the identification of *S. moniliformis* [11].

In our case, clinical clues of PJI prompted us to perform surgery on the TKA despite the fact that the cultures remained negative initially. We performed a DAIR procedure, based on steps agreed upon in our joint protocol for treating periprosthetic joint infections [12]. The protocol consists of open arthrotomy, sampling at least five deep tissue cultures, followed by extensive debridement with excision of possible infected necrotic tissues and synovectomy. Exchangeable prosthesis parts should be removed to provide optimal view and cleaning possibilities. However, this was not possible in our patient as the required insert was discontinued by the manufacturer. Parts of the prosthesis that remained in place were brushed with sponges to remove biofilm using povidon iodone solution. Finally, the joint was flushed with 6 L of saline using pulsed lavage. Gloves were changed and the knee was closed in layers. The surgical treatment was followed by antibiotic therapy, generally fourteen days of intravenous antibiotics, followed by ten weeks of oral antibiotics [12]. Based on multidisciplinary consultation with medical microbiologists and infectious diseases specialists, a course of intravenous ceftriaxone and oral doxycycline was chosen.

Two cases of rat bite fever PJI have been described before but with different treatment and outcomes. Stehle *et al*. (2003) have described a case of a patient with a history of bilateral TKA, who was successfully treated by oral antibiotics [10]. Smallbones *et al*. (2020) described a case of TKA with *S. moniliformis* infection, who was unsuccessfully treated by arthroscopy and hence in need of two-stage revision surgery [11]. It remains debatable whether patients could be treated purely with antibiotic therapy. We believe a DAIR procedure is superior to both antibiotic treatment, and arthroscopic lavage in the case of an infected arthroplasty with *S. moniliformis.* A DAIR procedure has a low morbidity and moderate to good chances of success if performed meticulously by experienced hands [12].

A possible limitation in this case was that no insert replacement could be done due to the fact that the manufacturer no longer produced the required insert. Also, the follow-up time was relatively short in the present case so recurrence may be possible in the future.

## Conclusions

In this case we have shown a good clinical response to the combination of a DAIR procedure and prolonged antibiotics for the treatment of a *S. moniliformis* periprosthetic joint infection. 16S rRNA gene sequencing was vital for the diagnosis. Future cases of S. *moniliformis* periprosthetic joint infection could be treated by the combination of DAIR and prolonged antibiotics.

## Data Availability

Relevant data are available on reasonable request.

## Abbreviations

PJI: Prosthetic joint infection
RBF: Rat bite fever
TKA: Total knee arthroplasty
DAIR: Debridement, antibiotics and implant retention
CRP: C-reactive protein
WBC: White blood cell count
MALDITOF: Matrix-assisted laser desorption/ionization time-of-flight mass spectrometry
PCR: Polymerase chain reaction
